# Evolution of the Public-Health Response to COVID-19 Pandemic in Spain: A Descriptive Qualitative Study

**DOI:** 10.3390/ijerph19073824

**Published:** 2022-03-23

**Authors:** Miguel Rodriguez-Arrastia, Manuel García-Martín, Ana Romero-López, Carmen Ropero-Padilla, Cristofer Ruiz-Gonzalez, Pablo Roman, Nuria Sanchez-Labraca

**Affiliations:** 1 Faculty of Health Sciences, Pre-Department of Nursing, Jaume I University, Av. Sos Baynat, 12071 Castello de la Plana, Spain; arrastia@uji.es; 2 Research Group CYS, Faculty of Health Sciences, Jaume I University, Av. Sos Baynat, 12071 Castello de la Plana, Spain; 3 Faculty of Health Sciences, Department of Nursing Science, Physiotherapy and Medicine, University of Almeria, 04120 Almeria, Spain; manugmartin@hotmail.com (M.G.-M.); anaromero.4@hotmail.com (A.R.-L.); cristofer4@outlook.es (C.R.-G.); pablo.roman@ual.es (P.R.); msl397@ual.es (N.S.-L.); 4 IMA S0082 Group, Hospital de Poniente, 04700 El Ejido, Spain; 5 Department of Gynaecology and Obstetrics, Torrecardenas University Hospital, 04009 Almeria, Spain; 6 Research Group CTS-451 Health Sciences, University of Almeria, 04120 Almeria, Spain; 7 Health Research Centre, University of Almeria, 04120 Almeria, Spain

**Keywords:** COVID-19, health personnel, nursing, patients, qualitative research

## Abstract

The capacity of hospitals and primary care centres has, rightfully, been at the centre of public and political debate on resource availability and control measures during the outbreak of COVID-19 and lockdown. Thus, the aim of this study is to describe the public and professional perceptions towards the evolution of the COVID-19 public-health response, in order to analyse and learn lessons for future health policies in similar situations in the future. A descriptive qualitative study was conducted through 41 in-depth interviews between January and June 2021. Twenty-one healthcare professionals and twenty service users participated in our study. The participants were recruited using purposive sampling. After our data analysis, three main themes emerged: (i) experiences during an unprecedented public health threat: the impact and challenges of early control measures, and outcomes for the public image of nursing; (ii) overcoming the impact of the outbreak on the healthcare system: professional coping strategies in the context of the pandemic, and institutional considerations in hospitals and primary care; and (iii) the efficiency of resource management during the outbreak: perceptions of professionals and healthcare users. Health providers and service users demand structural and organisational changes, as well as resource-optimisation strategies for front-line workers. Nurses need to be involved in decision making in order to provide evidence-based guidelines and ensure well-resourced and supported care practice.

## 1. Introduction

The global spread of COVID-19—the disease caused by severe acute respiratory syndrome coronavirus 2 (SARS-CoV-2)—has had profound ramifications on our personal lifestyles and professional practices, the global economy, and communities [1,2]. Notwithstanding the fact that the number of weekly cases and deaths has continued to decline, according to the World Health Organisation (WHO) (2021) [3], Europe has been the worst-affected region after the Americas, with countries such as Italy, France, and Spain among the worst-hit in the early stages of the pandemic [4]. From January 2020 to October 2021, there were almost 4.9 million confirmed cases of COVID-19 in Spain alone, with 86,701 documented deaths; this posed significant challenges for governments, policymakers and healthcare systems to provide an adequate and timely health response to the pandemic [5].

The pandemic has created uncharted territory in which improvising, reallocating, and making decisions about community care has become essential; this is due to a lack of data about the virus and potential approaches, as well as its rapid progression [6]. Given this new, unfamiliar context, front-line healthcare workers have been under enormous pressure to provide high-quality care, which has included, among other factors, fear of a greater risk of infection, an unknown future, and even a lack of appropriate personal protective equipment (PPE) [7]. This has compelled healthcare providers to make hasty and difficult decisions, such as allocating scarce resources evenly among patients, addressing their own physical and mental healthcare needs, or increasing long shifts to provide a stronger response [8]. In this regard, numerous studies have shown the effects of the handling and management of COVID-19 on healthcare workers, which have led to a high prevalence of compassion fatigue, emotional distress, and other mental-health-related issues such as anxiety, depression and burnout [9,10].

As the pandemic progressed and the number of COVID-19 cases increased, policymakers were forced to implement short-term measures at both health-system- and community-wide levels, in order to provide a rapid and effective response to reduce the spread of infection among the population [11]. Health facilities were strengthened in terms of equipment, technology, and personnel [12], while face-to-face consultations in primary care were reduced in favour of telephone attention, and some clinical procedures were purposefully delayed due to system-level changes [13]. Containment measures, on the other hand, were extended to the general public, including the general closure of all non-essential activities, social distancing, and other hygiene-related control measures [11]. However, the collapse of health centres and the increase in the number of health workers infected in their workplace have openly been at the centre of public and political debate regarding resource availability during the outbreak and lockdown [14]. According to data provided by the Carlos III Institute, more than 108 thousand infections among health and social-health personnel were reported in Spain by the end of 2021, with more than 335 thousand hospitalised and admitted into Intensive Care Units (ICUs) [15,16].

In the course of a pandemic, the combination of government leadership and public collaboration is critical, particularly when there is a high level of uncertainty about the risk and effectiveness of the control measures employed [17,18]. The public-health response to these efforts, however, has varied depending on context, age group, and the pandemic’s evolution, as well as the perception and image projected towards public service administration and the workers directly involved, including health professionals [19,20]. Different researchers have attempted to evaluate the public perception of pandemic control measures, as well as their impact on citizen collaboration [21,22]. In doing so, Seale and collaborators (2020) [23] concluded that inhabitants who had more trust in the government and authorities were more likely to follow the COVID-19 management measures. This issue must be tackled in Spain, which had one of the lowest scores of any country studied, with an average of 44.68 out of a possible 100, in COVID-SCORE, a questionnaire that provides a statistical score based on community impressions [24,25,26].

All of this information has led researchers to play an important role in the context of COVID-19, contributing to a better understanding of: how to manage, control, and prevent the pandemic through public-health responses [27]; the psychological impact of care disruptions [13]; the mental health impact on healthcare workers [10]; and the health risks of prolonged lockdowns among the wider population [11]. It is also necessary to consider the appropriateness of existing control measures and strategies from the perspective of workers and service users, given the wide variation in responses across countries and communities, and their impacts on the responsiveness of the health system [16]. The perceptions of users and healthcare professionals may have evolved as the epidemic progressed, allowing us to reflect on and describe these events for future similar scenarios. Thus, the aim of this study is to describe the public and professional perceptions towards the evolution of the COVID-19 public-health response in order to analyse and learn lessons for future health policies in similar and upcoming situations.

## 2. Materials and Methods

### 2.1. Design

This study used a qualitative descriptive design in order to obtain in-depth perceptions of the healthcare response to the COVID-19 pandemic among healthcare workers and service users [28]. This method contributed to increased flexibility in data collection and analysis, resulting in rich data and a detailed summary to the varied perspectives among the participants [29]. The study was performed from January to June 2021.

### 2.2. Participants

Participants were recruited in public hospitals and different primary care centres in Almeria (Andalusia, Spain) using purposive sampling, to provide the highest variability of participant experience with respect to the phenomenon under study [30]. The selection criteria among healthcare professionals included: (i) nurses, physicians or healthcare assistants who (ii) had more than or equal to 1 year of experience within the healthcare system; (ii) worked in different settings (e.g., hospital, community) on permanent or non-permanent contracts; and (iv) had professional experience during the outbreak. Likewise, healthcare service users who: (i) were 18 years old or older, and (ii) attended to the public healthcare system in the last 12 months, were also included. Sociodemographic characteristics are summarised in Table 1.

### 2.3. Data Collection

Researchers developed and agreed on an interview protocol (Table A1). The primary researcher approached each eligible participant and invited them to participate. Forty-one semi-structured interviews were conducted (21 healthcare professionals and 20 healthcare service users) at the University of Almeria by two researchers with training in qualitative research methods. Interviews were conducted in person, in accordance with safety protocols. Each interview was digitally audio recorded and lasted between 40 and 60 min. Data collection was continuously analysed until data saturation was reached, with 21 professional interviews and 20 user interviews. All transcripts were anonymised using the letters “P” (professional) and “SU” (service user) followed by the participant number. Participants were given the option to revise the recorded transcripts and read their transcriptions before beginning the data analysis process to ensure that their views were accurate.

### 2.4. Data Analysis

**Figure 1:**
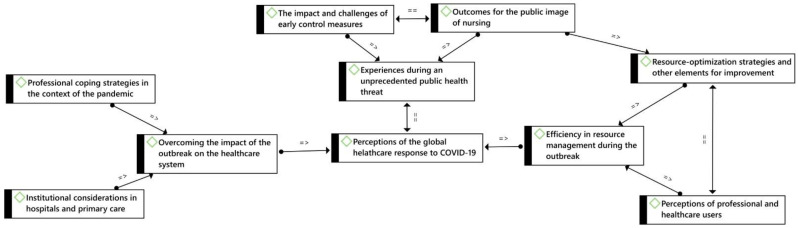
Conceptual map based on experiences and perceptions of the global healthcare response to COVID-19. = =: associated with; =>: cause of.

A thematic analysis was carried out using the ATLAS.ti v.9.0 software (Scientific Software Development GmbH, Berlin, Germany) [31], including the following steps: Data familiarisation was achieved by reading all the transcripts repeatedly and organising relevant data into meaningful codes, which were then classified into potential themes. Following that, these themes were reviewed by reading all the codes and the entire set of data to confirm thematic validity before defining and naming them and preparing a final report (Figure 1).

### 2.5. Ethical Considerations

The Ethics Committee of Nursing, Physiotherapy and Medicine Department at the University of Almeria accepted the study proposal (EFM 130/2021), which followed all of the criteria of the Declaration of Helsinki and its subsequent revisions. All participants were previously informed about the voluntary nature of their participation. Prior to starting, both users and professionals signed a consent form.

### 2.6. Rigour

Methods and findings are reported in line with the consolidated criteria for reporting qualitative (COREQ) research principles [32]. Furthermore, the thematic analysis was carried out independently by two researchers to ensure its validity and accuracy. In the event that their analyses differed, a third researcher was consulted to find consensus. The final results were accepted by all of the researchers.

## 3. Results

### Participant Characteristics

A total of 41 interviews were conducted, with 51.22% (n = 21) being healthcare professionals (10 nurses, 7 physicians and 4 healthcare assistants) and 48.78% (n = 20) being healthcare service users. The data were collected from January to June 2021. Overall, 63.41% of participants (n = 26) identified as female and 36.59% as male (n = 15). The ages of the participants ranged from 33 to 71 (48.22 ± 9.82 years). Qualitative analysis revealed three major themes, which are summarised in Table 2.

Theme 1: Experiences during an Unprecedented Public Health Threat

This theme addresses personal and professional experiences of the first COVID-19 control measures. Our results show the impact of uncertainty in their day-to-day lives, as well as how they faced and dealt with the different changes brought about by the pandemic situation. On the other hand, it also outlines the participants’ perceptions of how the COVID-19 pandemic has influenced the public image of healthcare workers, with the collective response of nurses emerging to describe front-line professionals who have taken personal risks to provide direct care to those infected with the virus.

Sub-theme 1.1: The Impact and Challenges of Early Control Measures

The majority of the participants, both professionals and users, stated that the early stages of the pandemic compelled them to make numerous changes in their personal and professional lives. In a personal context, changes in daily life, such as infection-control measures and being unable to see their families, were among the most significant factors that influenced service users more directly on a biopsychosocial level. Healthcare professionals, on the other hand, emphasised the emotional distress and compassion fatigue observed in fellow co-workers during the pandemic:

*“When the pandemic prevention and control measures were implemented, I had no idea what to do; I scrubbed myself every 5 min, and I had no clue what to do with my shoes, gloves, or facemask. For example, with my mother, we have been and continue to be incredibly cautious. It was a heartbreaking experience, but we were all compelled to stay at home for our own protection, unable to see or hug each other. It was and still is really hard.”*

SU-17

*“It was quite difficult for me to isolate myself because I may have had contact with a positive in COVID-19. I couldn’t even touch my little boys when I went home; I had to go to a special room to be isolated and frightened of infecting my own family. When we were first allowed to leave the house, I went to meet my parents with social distance, a mask, and so on, and I was surprised when my father said, “What is the point of living if I can’t hug my own daughter…?” It broke my heart”*

P-15

Sub-theme 1.2: Outcomes for the Public Image of Nursing

Many participants reported that the COVID-19 pandemic had a greater-than-ever positive impact on the visibility of nursing roles in the healthcare system. Most nurses did not perceive themselves as heroes in this regard; they considered it a necessary part of their job, as they are constantly putting themselves at risk of other contagious diseases. Indeed, some healthcare workers observed how this positive social recognition began to fade once the pandemic situations started to be stable:

*“At first, all healthcare professionals were viewed as superheroes for doing what they do every day. However, once the restrictions were relaxed, primary care providers became enemies because “the surgery was closed and we did not want to attend to anyone”. Even our hospital colleagues had a negative opinion of us, but what could we do? Were we the ones who made the decision? Primary care, I believe, has been essential to halting the pandemic”*

P-21

*“We are not superheroes. We have been doing the same thing our entire lives. I was moved by the clapping at first. I felt identified, but I was also certain that it wouldn’t last forever. What is more, once the pandemic was contained, the demands, rudeness, and aggression returned”*

P-4

Theme 2: Overcoming the Impact of the Outbreak on the Healthcare System

This theme highlights the professional participants’ perceptions of how the COVID-19 pandemic has changed their work when assisting service users, with feelings of frustration and a lack of human and material resources to provide proper and humane care. In this sense, some healthcare workers mentioned the institutional use of their worth as well as increased human and material resources, albeit acknowledging that most were insufficient, with recent graduates working in highly specialised services, or a general lack of training for new equipment.

Sub-theme 2.1: Professional Coping Strategies in the Context of the Pandemic

In this context, a number of healthcare professionals had to deal with not only workplace changes and challenges by implementing specific coping strategies—such as constant debriefing on COVID-19 policies, or professional support to reduce feelings of uncertainty and phycological distress—but also frustration when speaking with some users over the phone, as well as the fear of becoming infected:

*“We had regular meetings, especially during the first and second waves, to stay up to date on the COVID-19 guidelines at the unit. Yet, I feel that the most critical part for me has been the peer support from the beginning. I believe that if it wouldn’t have been for my colleagues, I would have broken down emotionally, knowing that I couldn’t provide the same level of care to everyone or, in other words, that I can’t do my job”*

P-7

*“For me, it was frustrating to have to chronic patients and older adults on the phone because we couldn’t see them in the primary care centres. Some of them are older adults and have some hearing problems, so all of this telenursing has been difficult for them at times. It was also tough to be in a dilemma when you needed to go to a patient’s home for a home visit because you are also a human being who is scared of becoming infected and exposing your loved ones”*

P-19

Sub-theme 2.2: Institutional Considerations in Hospitals and Primary Care

One of the most significant considerations for healthcare institutions to consider was related to the need for qualified and expert professional nurses in this pandemic context, rather than just employing a large number to overcome a staff shortage. Due to this, other professionals and service users stated the need for a sufficient staffing level in order to attend to users properly and in a timely manner, as well as to avoid the saturation of emergency departments:

*“True, they have doubled their staff and hired more people, but not just anyone will do in the ICU. We require experienced and well-trained professionals who are capable of getting the work done. In normal circumstances, we train newly graduated professionals in the ICU, but we don’t have time for that in a COVID-19 scenario and we don’t always know how to act”*

P-2

*“There isn’t enough staff at my primary care centre. If a physician retires, there will be no replacement for months, thus another physician will be required to care for those patients, causing the system to become overburdened... And when you try to make an appointment, you already know that it won’t be available in the next 7–10 days, so if you need something urgent, you end up going to the hospital because you can wait a week to be seen”*

SU-4

Theme 3: Efficiency in Resource Management during the Outbreak

This theme sheds a light on the importance of the flow of a wide-range of trustworthy and effective information among institutions, policymakers, managers, workers, and citizens. Additionally, healthcare professionals stressed the importance of fostering specific institution-centred training for COVID-19, as well as structural and organisational changes, in order to give a better response in these contexts.

Sub-theme 3.1: Perceptions of Professionals and Healthcare Users

One of the most frequently mentioned aspects of resource management during the pandemic by participants was the uneven control measures—largely in primary care settings, due to political divisiveness—and a lack of support from healthcare managers, among others:

*“Initially, there was a decrease in visits, but it got to the point where everything was urgent because people couldn’t go to the primary healthcare centre... Why weren’t professionals relocated to support these services? It was normal for service users to be annoyed, and for us to be disappointed... Why was that decision made? At the time, 80% of primary care has disappeared”*

P-14

Similarly, service users described the slow progress and higher-than-usual staff shortages they experienced when receiving care in any healthcare setting. Although they indicated that they were properly attended to by healthcare workers throughout the epidemic, what they noticed the most was a resource scarcity at primary care centres:

*“The professionals who have cared for me have always been great, but I’m not sure how they managed to accomplish so much with the resources they had. It was impossible to contact the primary healthcare centre, and when they did respond, they ended up referring you to the hospital. So, am I allowed to go to the supermarket and restaurants but not to the primary healthcare centre?”*

SU-20

Sub-theme 3.2: Resource-optimisation Strategies and other Elements for Improvement

A number of healthcare workers reiterated the importance of better training for several specific COVID-19 procedures, such as managing PPE or using the prone position with mechanical ventilation, for better resource optimisation; however, one of the most frequently requested strategies was better information flow and support between managers and workers:

*“It is true that we needed much more training and discussion meetings on how to trace patients infected with COVID-19, protocols, PPE, and so on. However, I believe that greater communication is what I have most missed, because a lack of information leads to confusion and unnecessary hostility among colleagues”*

P-17

Service users, on the other hand, reported poor resource management and discrepancies in infection control strategies. Service users identified trustworthy information flow between government, healthcare, and citizens as one of the most important strategies required, along with better resource-optimisation strategies to support healthcare workers, mainly in primary care:

*“I have seen a lot of differences in pandemic control measures from one location to the next. The strategies must be well-organised and well-coordinated. Above all, it was quite unequal in terms of resource allocation. They barely had anything in primary care settings, for example; they even had to wash their face masks!”*

SU-13

## 4. Discussion

The aim of this study was to explore public and professional perceptions of the evolution of the COVID-19 public-health response, in order to gain a better understanding of personal and professional impacts, as well as to address the resource-optimisation strategies implemented. After analysing our findings, it was found that almost all the participants—both professionals and service users—reported additional insecurities as a result of contradicting and dubious information about countermeasures, epidemiology, and a lack of efficient coping strategies or therapeutic mechanisms, which have been mostly overcome when compared to the initial number of COVID-infected people. While the coping strategies and physiological adjustments related to the COVID-19 outbreak have been widely discussed [10,33], this research reveals some intriguing findings regarding the impact of the pandemic on the public image of nursing and professional identity, as well as suggesting potential resource-optimisation strategies for the future. To the best of our knowledge, this is the first study to explore the intertwined experiences of both professionals and service users in order to delve into personal and professional changes, coping strategies, and resource-optimisation strategies from a qualitative perspective.

Our findings showed that the initial stages of the COVID-19 pandemic were associated with greater changes in work and lifestyle among our participants [34,35]. Some of these changes included social distancing, self-isolation, and quarantine of those who had contracted or had been at risk of contracting the novel coronavirus; these have been shown to cause emotional and mental health problems, as well as less healthy eating habits; a lack of good-quality sleep; a significant decrease in well-being and physical activity; and an increase in sedentary habits [36]. In this vein, front-line healthcare professionals in certain settings—such as primary care, emergency departments, and critical care departments—have had no choice but to continue their care work while balancing the needs of patients with their own and their families’, which, according to our findings, may have resulted in vicarious trauma and compassion fatigue among professionals [37]. One possible explanation for this could be the scarcity of knowledge available in the early stages of the pandemic, as well as the desire of these professionals to alleviate the suffering of others; this may have outweighed their ability to provide high-quality care in some circumstances and required them to undertake far-reaching ethical and moral decisions [38]. The findings of this study, however, also indicate that the majority of participants used some coping strategies to overcome mental health challenges, with problem- and emotional-based coping strategies being more common among professionals, and avoidant coping strategies more common among service users. According to previous findings [33,39], this could indicate that professionals, particularly front-line nurses, are keen to feel in control of stressful situations and foster moral courage despite experiencing stressful events; however, this could also have contributed to the archetype of heroic nurses during the pandemic [40].

Front-line nurses have been culturally portrayed as being proud of their role as the last line of defence during the initial stages of the pandemic [41]. While the media and service users frequently used the concepts of sacrifice and selflessness to describe nurses, most of these professionals felt that this discourse resulted in the normalisation of some unacceptable risks, such as leaving older patients to die alone or taking personal risks as a moral act [42]. Social recognition rituals such as clapping represented a cultural reward for healthcare professionals [43]; however, many of these workers described this public attribution as a discursive pattern, shifting from perceiving healthcare professionals as outstanding and valorised to mundane and unappreciated as the hardest stages of the pandemic are overcome. Indeed, the voices of these professionals suggested that the hero discourse failed to materialise longer-term systematic changes and long-standing policy changes into the already eroding and unsafe working conditions in healthcare institutions, leading these workers to perceive themselves as nothing more than hardworking, productive, and expendable subjects [44].

Despite the general public satisfaction with the performance of healthcare professionals, some of our participants expressed their scepticism and concern as the uneven public health measures, lack of personal protective equipment, and scarce organisational staffing persisted in the context of the COVID-19 outbreak [7]. Certainly, a number of participants mentioned shortfalls in resource management not only in emergency and critical care departments, but also, and especially, in primary care. Whereas other unmeasured medical, economic, and social indicators of vulnerability may exist in this area of care [45], the novel coronavirus—along with its associated economic downturn—has disproportionately affected and limited access to primary care, despite knowing that this area has been absolutely essential for contact tracing, testing and control of COVID-19 [46]. These findings further support the idea that government entities, health organisations and nurse leaders need to be better prepared to allocate available resources and develop resource-optimisation strategies, as well as other elements, to support their already burdened staff and retain highly trained professionals in their services [12]. Some short- and long-term strategies that could be implemented include structural and organisational changes, such as ensuring that staff do not work longer than safe working hours, or evidence-based pre-established criteria for allocating resources (e.g., beds, medication, PPE, etc.), but also other elements including integrating self-care strategies into working daily practice [47]. In this sense, nurse managers and leaders could indeed support others and serve as role models for good self-care in order to promote good mental health and well-being [48]. It is important for nurse managers to be available and to create opportunities for moral reflection and ethical discussions with fellow front-line nurses in order to mitigate moral distress, especially during times of complexity such as during the COVID-19 pandemic. It is also important to ensure that self-care plans for healthcare providers are available to help in coping with anxiety and fears that may arise when caring for these kinds of patients [49].

### 4.1. Limitations

There were also some limitations to this study. It is acknowledged that due to the qualitative nature of this study, our findings may not be representative of the experiences of all professionals and service users. Future research should look into the experiences and perceptions of other groups, such as allied healthcare workers, midwives, and students, who may have had similar experiences and could provide a broader perspective on the issue. Future research will have to investigate to what extent COVID-19 has affected users, relatives and professionals in other settings such as private hospitals or nursing homes. Likewise, other service users with special needs and disabilities could benefit from being included in future studies. This study, on the other hand, offers a valuable insight into the perspectives of both front-line personnel and active service users from different settings. These findings may not only contribute to better patient care, especially in difficult times, but also provide managers, policymakers, and organisations with an opportunity to better understand and support their professionals in an effort to increase engagement and retention rates.

### 4.2. Relevance to Clinical Practice

In view of nursing relevance in difficult contexts such as the COVID-19 pandemic, nurse managers must be aware of their needs. Front-line nurses have proven to be resourceful, but they are exhausted and fatigued; hence, funding and support are required to continue normal healthcare services, notably in primary healthcare, while preventing moral distress and compassion fatigue. Nurse managers have the opportunity and the moral responsibility to involve front-line nurses in decision making in order to provide evidence-based guidelines and ensure well-resourced and supported care practice.

## 5. Conclusions

Our findings outline the critical importance that the healthcare workforce, particularly nursing personnel, has had in the global response to the COVID-19 pandemic. While problem- and emotion-based coping strategies were most commonly adopted by healthcare professionals throughout the pandemic, this study suggests that moral distress and compassion fatigue are still persistent as a result of a lack of support from healthcare managers and supervisors. Both health providers and service users demand structural and organisational changes in order for long-term systematic changes and long-standing policy changes to become a reality. These changes include evidence-based pre-established criteria for allocating resources in hospitals (and particularly in primary care settings), trustworthy information flow, and self-care strategies for front-line professionals to promote mental health and well-being, as well as retaining highly trained professionals.

## Figures and Tables

**Table 1:** Summary of each participant’s characteristics.

**Professionals**					
**Code**	**Experience (Years)**	**Age**	**Sex**	**Job Position**	**Clinical Area (Department)**
P1	6	34	F	RN	Intensive Care Unit
P2	12	37	F	RN	Intensive Care Unit
P3	20	42	F	RN	Emergency
P4	28	50	F	RN	Emergency
P5	23	46	F	RN	Emergency
P6	17	38	F	RN	Intensive Care Unit
P7	19	35	M	RN	Intensive Care Unit
P8	22	46	F	RN	Emergency
P9	28	52	M	Phys	Emergency
P10	24	45	F	Phys	Intensive Care Unit
P11	25	49	M	Phys	Intensive Care Unit
P12	30	55	F	Phys	Emergency
P13	6	33	F	HCA	Intensive Care Unit
P14	10	37	M	HCA	Emergency
P15	32	57	F	HCA	Intensive Care Unit
P16	36	59	M	Phys	Primary Healthcare
P17	34	55	M	Phys	Primary Healthcare
P18	13	49	F	Phys	Emergency
P19	33	57	F	HCA	Primary Healthcare
P20	17	39	F	RN	Primary Healthcare
P21	14	35	F	RN	Primary Healthcare
**Service Users**					
**Code**	**Age**		**Sex**	**Clinical Area (Department)**	
SU1	36		M	Emergency	
SU2	44		M	Emergency	
SU3	41		F	Emergency	
SU4	53		M	Primary Healthcare	
SU5	47		F	Emergency	
SU6	39		F	Emergency	
SU7	40		F	Emergency	
SU8	54		M	Primary Healthcare	
SU9	58		M	Primary Healthcare	
SU10	62		F	Emergency	
SU11	42		F	Primary Healthcare	
SU12	48		M	Emergency	
SU13	47		F	Primary Healthcare	
SU14	56		M	Primary Healthcare	
SU15	60		M	Emergency	
SU16	45		F	Emergency	
SU17	66		F	Emergency	
SU18	50		F	Emergency	
SU19	68		F	Primary Healthcare	
SU20	71		M	Primary Healthcare	

F: female; M: male; RN: registered nurse; Phys: physician; HCA: healthcare assistant.

**Table 2:** Themes, sub-themes and representative quotes.

Main Themes	Sub-Themes	Representative Quotes
Experiences during an unprecedented public health threat	The impact and challenges of early control measures	*“For me, the most challenging part was the quarantine. I was alone at home because I live alone, and the only way I could communicate with my family and friends was via videocalls. That was the only thing that kept me alive”* SU-2
		*“I recall a lot of anguish and fear at the hospital, seeing colleagues get infected and even caring for them. It was very hard, emotionally speaking. I remember those times with grief and sorrow”* P-1
	Outcomes for the public image of nursing	*“Seeing how hard the health workers, particularly nurses, had to work made me realise how important their job is. They have been through a lot and yet, they never quit caring for others”* SU-7
		*“I feel the pandemic has increased the visibility of professional nurses. Many of us would have said, “If I were you, I am not sure if I would do it”. They gave everything they had and then more. I am really proud of the care they showed me and my loved ones”* SU-15
Overcoming the impact of the outbreak on the healthcare system	Professional coping strategies in the context of the pandemic	*“We all started to react after the first signs of the pandemic. Peer support was constant; there was always someone around to offer encouragement when things became tough. Despite protocols being changed frequently, we had meetings every now and then to discuss how we should respond. We felt that we weren’t about to given appropriate care sometimes, and these meetings gave us some light amongst all the chaos”* P-10
		*“When things began to return to normal, I was concerned because all of the waiting rooms became overcrowded again, and our fear was that if we continued like that, the pandemic would reach high contagious rates again and more people will die”* P-12
	Institutional considerations in hospitals and primary care	*“It is true that they tried to hire more staff, but I believe it has been more of a barrier than a benefit on many occasions. Is it really investing in staff if you engage a new colleague with no experience in specialised services such as ICU with COVID-infected patients?”* P-13
		*“We need more staff; we can’t have three physicians where there should be five or six, and users are aware of this because when they try to book an appointment, they sometimes have to wait a week, and if they need to be attended for any disease, they can’t wait a week, so they eventually go to the hospital, to the emergency unit; and of course, the emergency unit becomes overcrowded, which is not right, but what can we do about it?”* P-16
Efficiency of resource management during the outbreak	Perceptions of professionals and healthcare users	*“* *We would have appreciated further support from our managers. It would have been great if they had stopped by and asked what we needed. We were constantly late and sloppy, but luckily, we could coordinate ourselves”* P-8
		*“Even though it was over the phone, I was properly taken good care of. In general, they are professionals who do their jobs well, but they are limited by a significant lack of resources. They are undervalued and under-supported, particularly in services such as primary care”* SU-11
	Resource-optimisation strategies and other elements for improvement	*“I think our supervisors should have provided more information to avoid confusion, such as more clinical sessions, PPE use, etc. I believe we would have felt more confident working if we would have had more support”* P-5
		*“I feel that resource distribution strategies should be more consistent so that supplies do not run out. This has been extremely lacking all through the pandemic. Not to mention the importance of looking after the professionals. Yet, I should admit this was a difficult scenario for any government in the world”* SU-12

## Data Availability

The data that support the findings of this study are available on request from the corresponding author. The data are not publicly available due to privacy or ethical restrictions.

## Appendix A

**Table A1:** Interview protocol.

Stages of the Interview	Topics	Examples	
Introduction	Purpose of the study	My colleague and I are participating in a study to better understand the global healthcare response to COVID-19. We believe your experience may be useful to implement measures to improve healthcare delivery in similar scenarios	
	Objectives	Carry out and publish research based on your experiences in healthcare responses to the COVID-19 pandemic	
	Ethical considerations	Our conversation will be recorded solely for research purposes in order to carry out our research. Just keep in mind that participation is entirely voluntary, and you can opt out at any time. Everything said during this interview will be kept strictly confidential, anonymised, and available only to the research team	
	Verbal and formal consent	Granted if the participant agreed verbally and signed the formal consent	
Development	Interview grid	Healthcare workers	Service users
		Could you please describe how you lived and what your feelings and thoughts were during the early days of the pandemic?	
		Please, tell me a little bit about how the pandemic impacted your work; could you describe the changes you saw?	
		Could you describe what measures or resources have been made available to address the pandemic from your company or institution?	
		What aspects, habits, or behaviours do you believe have changed in your day-to-day job on a personal level?	
		How do you consider preventative training was handled in your workplace during the pandemic?	
		What were your thoughts and feelings about the everyday social recognition during the beginning of the pandemic?	
		Do you believe there has been a shift in this recognition? Why?	
Closing	Final questions	Do you have anything else to add that might be relevant? Anything to clarify before we end?	
	Acknowledgements	Thank you for your time and interest. Certainly, your statements will be useful for the research	
	Considerations	Please, let us know if you need anything else	
		Once the study is finished, we will send you a copy	
Translation	Retro-translations	Interview statements will be translated by one bilingual researcher to English. Then, another bilingual researcher will back-translate them to Spanish and compare them with the original transcripts to maintain their accuracy	
